# Variance in multi-blade induced lightning overvoltages among different wind farm topologies

**DOI:** 10.1371/journal.pone.0308449

**Published:** 2024-09-05

**Authors:** Fady Wadie, Ali Saeed Almuflih, Z. M. S. Elbarybary, Tamer Eliyan

**Affiliations:** 1 Faculty of Engineering, Mechatronics and Robotics Engineering Department, Egyptian Russian University, Badr City, Egypt; 2 Department of Industrial Engineering, College of Engineering, King Khalid University, Abha, Saudi Arabia; 3 Department of Electrical Engineering, College of Engineering, King Khalid University, Abha, Saudi Arabia; 4 Faculty of Engineering at Shoubra, Department of Electrical Engineering, Benha University, Cairo, Egypt; 5 Department of Electrical Power and Machines Engineering, The Higher Institute of Engineering at El-Shorouk City, Alshorouk Academy, Cairo, Egypt; University of Cagliari, ITALY

## Abstract

The impact of the topological formation of wind farms upon the lightning induced overvoltages injected into the grid was not covered earlier in literature. However, this topic is highly important to be investigated to allow the usage of the most reliable topology against lightning strikes. For such reason, the paper investigates this point with consideration of most damaging cases as lightning strikes to multi-blades. The testing used ATP software for four main topologies, radial, single-sided ring SSR, double sided ring and star topology. The features defining the similarities in response and the variance range between these topologies were recorded and analyzed. The multi-blade strikes gave an expected increase of 15% to 100% in the injected overvoltage to the grid for all topologies. The star topology showed the most reliable performance by allowing the least injected overvoltage to the grid. The percentage of reduction in the magnitude of the injected overvoltages reached 50.78%, 66.07% and 89.04% for SSR, DSR and star topology respectively with respect to radial topology. Recommendation was provided for design engineers to consider star topology during design phase in terms of more reliable lightning protection.

## Introduction

The transformation to green energy power generation has always faced the challenge of the random nature for natural sources for renewable energy [1–9]. Such natural sources have mandated specific locations for renewable energy resources to be feasible for implementation, which in turn endangered these sources by being exposed to hazardous weather conditions. Specifically, wind farms have been under the impact of lightning strikes requiring protective measures to be taken for their protection [10–13]. The most exposed element within wind farms, is the blades of their turbines. That is due to being exposed with a very long height in exposed areas [14–18]. A direct lightning strike on the tip of one the blades of the wind turbine will lead to the incidence of a surge that propagates across the tower structure causing a gigantic rise in potential across tower elements [16–23]. The impact of these strikes upon the different elements of the wind farm have been under study with researchers focusing upon the impact across tower structure [15–18] or across the grounding system [15,24,25] and across the remaining electrical network of the wind farm [26–30]. However, researches in [26–30] focused upon the lightning induced within wind farms of radial topology only and other topologies were not considered. For this reason, the paper intends to focus on impact of the lightning induced surges in different wind farm topologies. Four topologies are considered in this research; radial, single-sided ring (SSR), double-sided ring (DSR), and star topologies [31,32]. This would allow the viewing of the degree of variance of the impact of lightning strikes from one topology to another. Hence, it could be defined which the wind farm topologies that provide more reliable response in terms of the lightning induced surges.

A second aspect that should be considered is the type of the lightning strikes including positive, and negative lightning strikes [33–36]. However, not only the type of the strike affects the resulting induced overvoltages, but also the possibility of hitting multi-blades of the turbine. [10,16,17]. The strike which hit multi-blades could be initiated by scenarios including the sweeping of lightning flash from one blade to another as a result of the blade’s rotation, branching of the lightning strike to reach more than one blade or subsequent lightning strikes to other blades. Even though the occurrence of a strike that hits multi-blades has a low probability but it still relatively dangerous once it happens. That’s due to the significant damages to the blades and economic loss are greater than those under the lightning strike to single-blade [10,16]. Hence, this study will consider the dual impact of changing the type of wind topology and the possibility of striking a multi-blade upon the resulting overvoltages. ATP/EMTP simulation platform was used in this study. The main contributions of the paper include:

1. Investigating a rare topic in literature which is the impact of changing the topology of the wind farm upon the magnitude of the resulting lightning induced overvoltages
2. Extending the investigation to include the variance in results for lightning strikes and possible strikes to multi-blades which represent highly damageable scenarios.
3. Defining the levels of variation in the magnitude of resulting overvoltages for different wind farm topologies under different multi-blade scenarios.
4. Evaluating the response within each topology under extreme multi-blade conditions allowing to present conclusions that would assist design engineers in selecting the most suitable wind farm topology during the design phase.

The rest of this paper is arranged in the following order. The data and structure of the system selected to be under investigation, it’s modeling process upon ATP/EMTP are sequentially presented in sections of system under study and modeling of the system. The results of the simulation in different topological cases and under different striking conditions are presented in simulation results section and later analyzed in analysis and discussion section. Finally, conclusions are drawn in conclusions section.

## System under study

**Fig 1:**
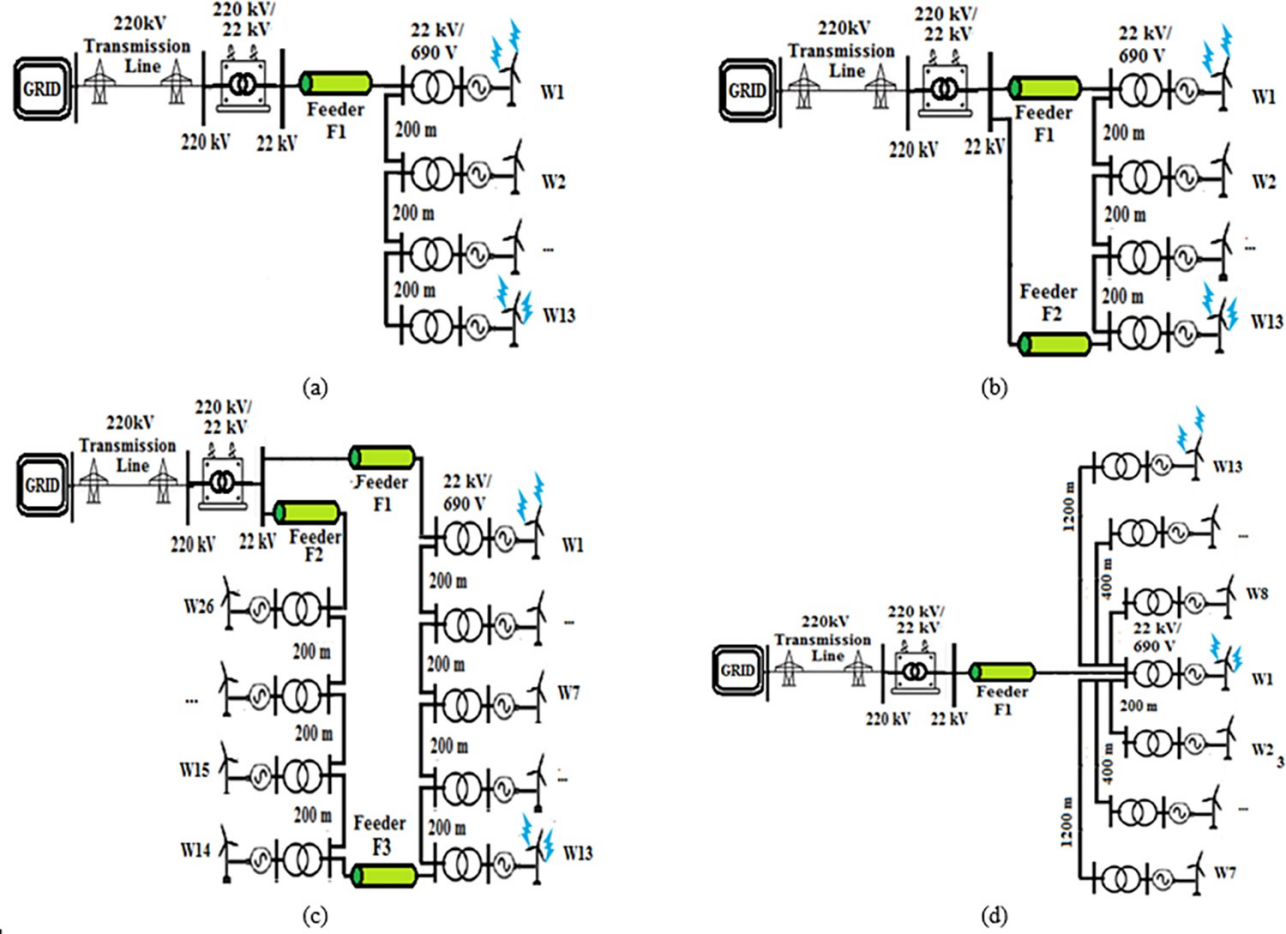
Topologies of wind farm (a) Radial (b) Single sided ring (b) Double sided ring (d) Star.

**Fig 2:**
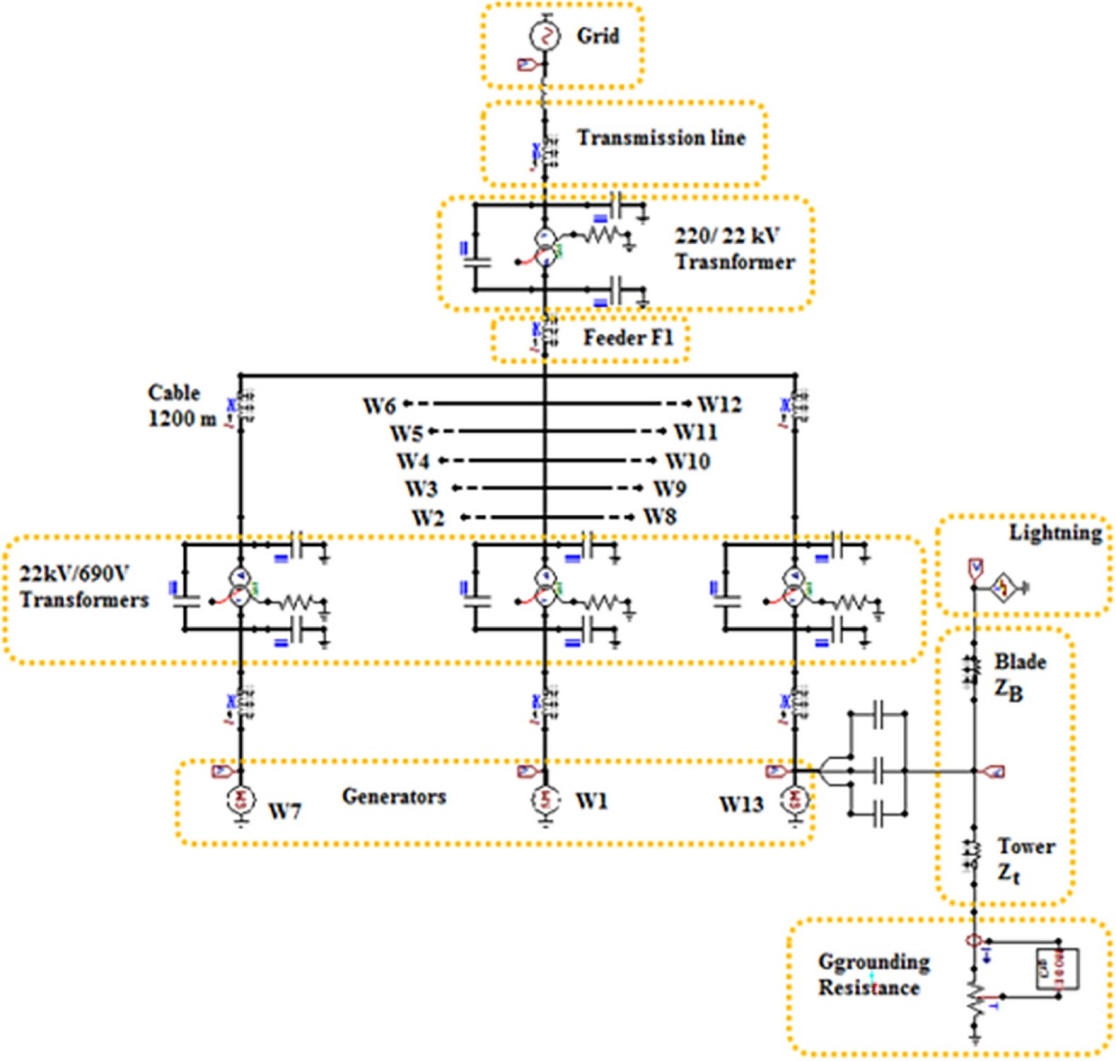
Circuit diagram of star topology using ATP software.

The system used for testing was based on the data from a real 550 MW wind farm system located in Zaafrana, Egypt. The system consists of 700 wind turbines which were assumed to be identical with each of them connected to a 1 MVA 690 V/22 kV-transformer. 200 m cables were used to connect each two turbines following each other in sequential order. A 220/22 kV substation ties the farm to the grid. The data of the elements of the introduced system will remain unchanged but the topology of the system will be changed to examine the variance in their response during lightning strikes as previously defined to be one of the aims of the paper. The testing topologies include radial, SSR, DSR and star topologies as shown in Fig 1. The lengths of the feeders are as follows; F1 = 8 km for all topologies. F2 = 10.4 km in SSR topology and 6.5 km in DSR topology, F3 = 1 km. The lengths of the cables will be 200 m for all topologies except for star topologies due to its design nature that require the cables connected to each turbine to be of different lengths. The lengths of cables for star topology follows the next pattern; 200 m for W2 and W8, 400 m for W3 and W9, 600 m for W4 and W10, and so on till W7 and W13. The modifications done is based upon those presented in [37]. The modeling of the elements of the system is discussed in the next section. The circuit diagram used for modeling star topology on ATP software is shown in Fig 2.

## Modeling of the system

### Modeling of the wind turbine

#### Tower and blades of the wind turbine

The physical formation of blades of tower body of the wind turbine was used build its model. Therefore, the surge impedances of the tower and blade were driven from cylindrical and conical equations of the electromagnetic field theory as given in (1) and (2) respectively [38,39].


$$Z_{t}=60\;ln(\frac{\sqrt{2}H}{r})$$ (1)

$$Z_{B}=60\;ln(\frac{2L_{b}}{r_{b}})$$ (2)

Where “r” and “H” are the radius and height of the base of the tower respectively *r*_*b*_. and *L*_*b*_ are the radius of down conductor and the length of the blade. The Modeling of the tower and blade could be more detailed as those provided in [1–4,10–13], however these research works were intending to study the potential rise in the grounding system and the impact upon the body of the tower. Therefore, the detailed modeling for them was justified, unlike this paper where the main focus is the transmitted overvoltage to the electrical network and hence, the usage of approximated modeling justified as earlier used in [38,39].

#### Grounding resistance of the tower

The modeling of the grounding resistance of the tower during lighting strikes requires the consideration of its value during transient response of the strike. The event of the lightning strike injects excessive amount of energy into the ground which ionizes it and changes its value. This variation of the resistance could be represented as a non-linear resistance (Rg) as given in (3) and (4) [40,41]. A mathematically controlled non-linear resistance model was implemented on ATP/EMTP using non-linear TACSRES component.

$$R_{g}=\left\{ \begin{array}{l} R_{go}\;(I<I_{o})\\ \;\frac{R_{go}}{\sqrt{1+\frac{I}{I_{o}}}}\;(I>I_{o}) \end{array}\right.$$ (3)

$$I_{0}=\frac{\rho_{s}E_{o}}{2\pi R_{go}^{2}}$$ (4)

where *R*_*go*_, *R*_*g*_, are the values of the grounding resistances of the tower during normal operating conditions and lightning strikes respectively, “I, I_o_” are the currents flowing into the grounding resistance and the minimal current value required for the soil to ionize respectively with calculation of I_o_ given in (4). ρ_s_ and *E*_*o*_ are frequency dependent soil resistivity and the gradient of soil ionization respectively. The values of E_o_, ρ_s_ and R_go_ were used as 400 kV/m, 1000 Ω.m and 10 Ω respectively according to [41].

#### Generator of the wind turbine

A 690 V synchronous generator model was used with leakage reactance of 0.1 H [37]. The body of the nacelle and the generator form a stray capacitance between them that has a value of few nano-farads and was set to 10 nF in this study [38,39]. The inclusion of this capacitance in the model is very important as it allows the simulation of the inducted overvoltage into the electrical system of tower from the lightning surge propagating through the tower body.

*Transmission lines*, *feeders and cables*. Transmission lines were modeled using frequency-dependent line models such that the parameters used followed the data given in Table 1 [37]. Similar approach was used for cables with consideration of their lengths as previously described in system under study section.

**Table 1:** Parameters of transmission line.

	Zero sequence parameters	Positive and negative sequence parameters
Resistance (Ω/km)	0.13	0.03
Reactance (Ω/km)	0.83	0.306
Susceptance (mS/km)	2.3	3.25

#### Power transformers

Transformers were modeled using frequency-dependent transformer component upon ATP. The modeling of the stray capacitance between each winding and the ground between the windings with each other was implemented by using capacitive elements connected across transformer [19,37].

#### Lightning strike

Lightning strikes was modeled as an impulse surge current source that is injected to the location of the strike. The formulation of lightning surge according to Heidler function is given in (5) and (6) [26,33].


i(t)=Ioη.(tτ1)n1+(tτ1)nexp(−t/τ2) (5)

$$\eta =\exp(-(\frac{\tau_{1}}{\tau_{2}})(n\frac{\tau_{2}}{\tau_{1}}{)}^{\frac{1}{n}})$$ (6)

Where *I*_*o*_ is the peak value of lightning impulse; *τ*_1_ and *τ*_2_ are the rise and tail times of the impulse respectively, n is the exponent factor and *η* is the peak value correction factor. The values of the parameters of the lightning impulse depend on its type. Two types are studied in this work; positive, and negative according to their standard parametric data as follows:

1. Negative lightning strike of 1/200 μs based upon the IEC 61400–24 standard [42].
2. Positive lightning strikes of 10/350 μs according to the IEC 61400–24 and IEC 61643–11 standards [41,42].

## Simulation results

The system described in system under study section was modeled as stated in modeling of the system section and lightning strikes were created to hit the blades of the wind turbines. To consider the range of possibilities of the lightning induced overvoltages, two extreme cases were chosen for the defining the turbine selected to be hit by lightning. These cases include the hit location to be at the blades of first the tower closest to the grid or the one farthest to the grid. For each case, considerations of single, double and triple blade hit by lightning strikes were taken during the testing process. The results focused on the overvoltages propagating from blade to tower and then to the 220 kV/22 V transformer connected to the grid will be examined. That to evaluate the amount of damage that will reach the wind farm and the grid. The previous sequence was repeated for different wind turbine topologies to allow a comparison between the results of these cases and define the impact of changing the wind farm topology upon the results.

### Radial topology

**Fig 3:**
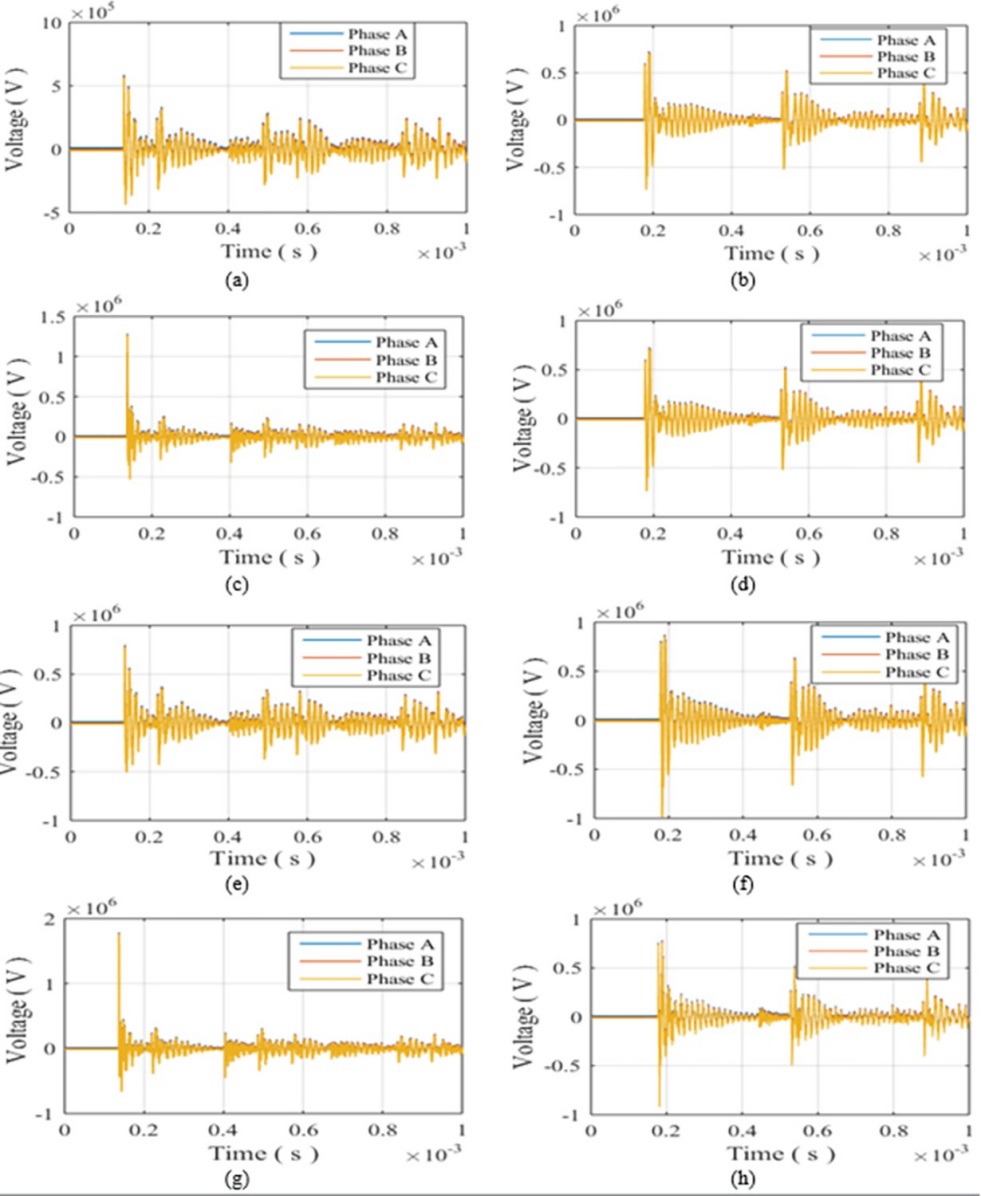
Overvoltages at low-voltage bus of the grid connected transformer in radial topology for cases (a) R-5 (b) R-6 (c) R-7 (d) R-8 (e) R-9 (f) R-10 (g) R-11 (h) R-12.

The first step is defining, the strike locations which will be W1 as the closest turbine to the grid and W13 as the farthest one. For each of them, single positive and negative strikes will be applied at first. Then double-blade and triple blade hit by positive and negative strikes will be tested for both turbines. The peak values for positive strikes are relatively larger than negative therefore for positive strikes peaks of 200, 150 and 100 kA are considered while for negative 100, 75, 50 kA are considered [10]. The peak values of strikes hitting double blades and were considered equal, while in triple-blade were considered unequal as in lightning branching scenario. In such way of peak selection, a consideration for different peak scenarios is simulated. The parameters of the tested cases are defined in Table 2 with cases given numbers from R-1 to R-12 such that initial letter R stands for radial. The overvoltage induced at each cases upon the blade tip, the top of the tower, the generator within the turbine and the 22 kV bus of the 220/22 kV transformer are recorded in Table 2. The maximum overvoltage at the low voltage bus of the grid transformer for case R-5 to R-12 are shown in Fig 3.

**Table 2:** Maximum overvoltages recorded for radial topology.

Case name	Number of blades and the turbine hit	Type and parameters of lightning	Maximum Voltage at (MV)			Maximum Voltage at low voltage bus of grid-connected transformer (kV)		
			blade	Top of tower	Generator	Phase A	Phase B	Phase C
R- 1	1 blade of W1	Positive 200 kA, 10/350 μs	24.45	14.19	4.52	472.84	462.47	450.89
R- 2	1 blade of W13	Positive 200 kA, 10/350 μs	24.45	14.14	4.49	679.85	669.74	657.91
R- 3	1 blade of W1	Negative 75 kA, 1/200 μs	13.39	8.82	2.79	930.03	919.59	908.07
R- 4	1 blade of W13	Negative 75 kA, 1/200 μs	13.39	8.71	2.67	536.12	525.99	514.81
R- 5	2 blades of W1	Positive 150 and 150 kA, 10/350 μs	18.57	17.33	4.70	585.60	575.15	563.63
R- 6	2 blades of W13	Positive 150 and 150 kA, 10/350 μs	18.48	17.25	4.68	729.99	726.29	738.07
R- 7	2 blades of W1	Negative 75 and 75 kA, 1/200 μs	11.15	10.93	2.95	1284.00	1273.50	1262.00
R- 8	2 blades of W13	Negative 75 and 75 kA, 1/200 μs	10.96	10.79	2.85	646.03	656.21	667.98
R- 9	3 blades of W1	Positive 200,150 and 100 kA, 10/350 μs	24.47	21.84	5.48	799.29	788.84	777.33
R- 10	3 blades of W13	Positive 200,150 and 100 kA, 10/350 μs	24.48	21.74	5.42	966.46	976.63	988.41
R- 11	3 blades of W1	Negative 100,75 and 50 kA, 1/200 μs	14.25	13.86	3.38	1784.30	1773.80	1762.30
R- 12	3 blades of W13	Negative 100,75 and 50 kA, 1/200 μs	14.01	13.72	3.29	898.67	908.84	920.62

From the results shown in Table 2, several features could be defined for lightning induced overvoltage reaching the grid side from radial topology:

**F1**. The induced overvoltages reaching the transformer connected to the grid are higher for negative strikes than positive strikes.
**F2**. The highest magnitude of the injected overvoltage reaching the grid transformer are from lightning strikes the farthest tower from the grid for positive strikes and the opposite for negative strikes.
**F3**. Double blade and triple blade hits generally increase the induced overvoltage reaching the transformer connected to the grid, and when compared to the single strike overvoltage of the same case it is found a percentage of increase from 20% up to 94%.

The previous features are further analyzed and discussed in analysis and discussion section. The same testing procedures are applied for remaining topologies in this section to if these features apply also to them or not, and more importantly to compare the variance in results between these topologies.

### Single sided ring topology

**Fig 4:**
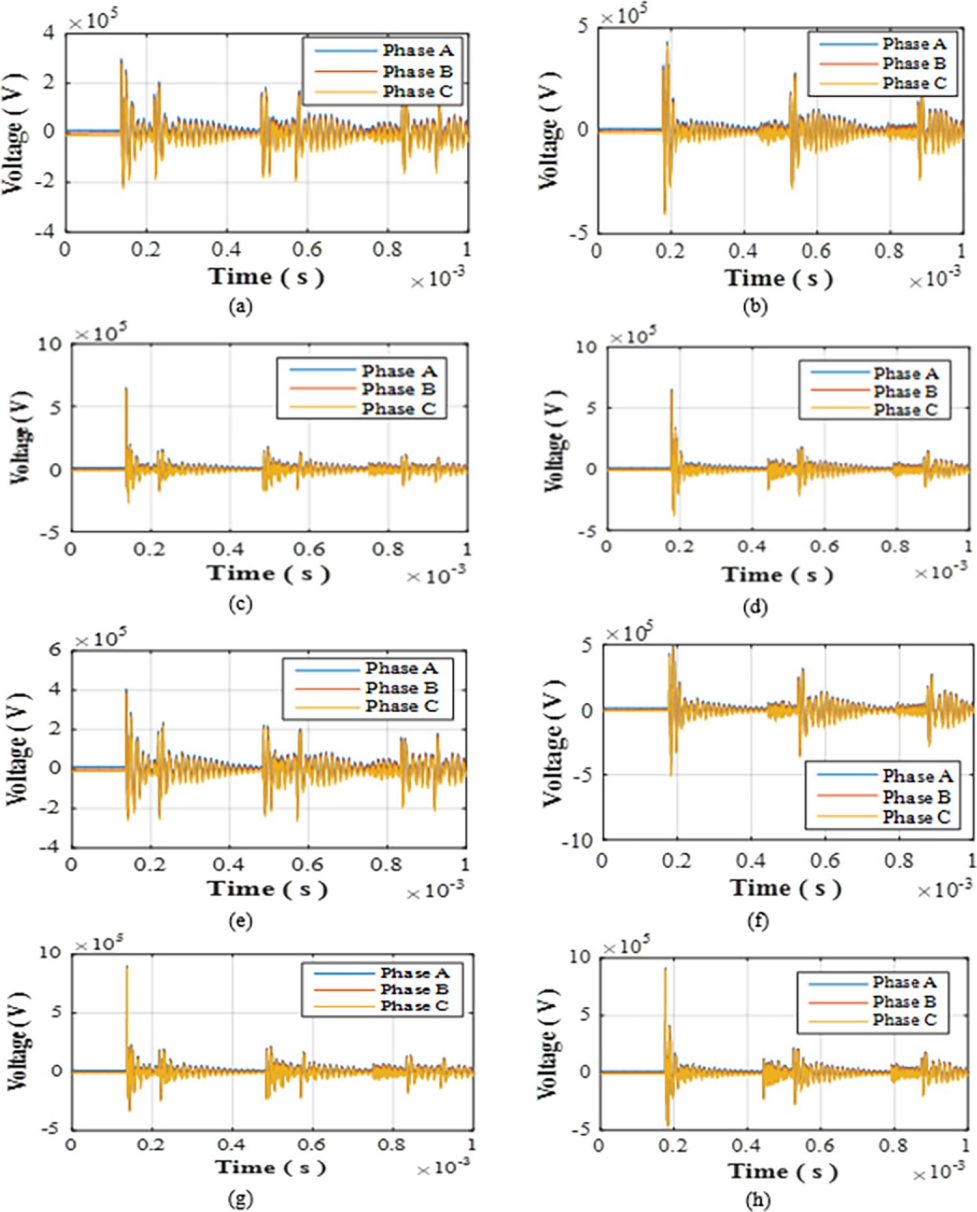
Overvoltages at low-voltage bus of the grid connected transformer in SSR topology for cases (a) SSR-5 (b) SSR-6 (c) SSR-7 (d) SSR-8 (e) SSR-9 (f) SSR-10 (g) SSR-11 (h) SSR-12.

The testing procedures were re-done for the SSR topology. The parameters of the tested cases are given in Table 3 with cases notated as SSR-1 to SSR-12. The maximum overvoltage at the low voltage bus of the grid transformer for case SSR-5 to SSR-12 are shown in Fig 4. The results show that the features previously defined for radial topology also applies for SSR-topology. For feature F3, the double and triple blade hits allowed an increase in overvoltage in range of 23.3% as in Phase A of SSR-5 to 97.8% as in phase C of SSR-12 when compared to single strikes of the same parameters. The comparison between cases of radial and their counter parts in SSR topology shows a general reduction in the induced overvoltage. The reduction percentage ranged from 11.75% for phase A from SSR-4 when compared to R-4, to 50.78% for phase C of SSR-5 when compared to R-5. Hence, a new feature is added to those mentioned in radial topology subsection as follows:

**F4. Induced lightning overvoltage reaching the transformer connecting to the grid are generally reduced in SSR topology than radial topology by range from 11.75% to 50.78%**.

**Table 3:** Maximum overvoltages recorded for SSR topology.

Case name	Number of blades and the turbine hit	Type and parameters of lightning	Maximum Voltage at (MV)			Maximum Voltage at low voltage bus of grid-connected transformer (kV)		
			blade	Top of tower	Generator	Phase A	Phase B	Phase C
SSR- 1	1 blade of W1	Positive 200 kA, 10/350 μs	24.45	14.19	4.52	242.75	232.36	220.78
SSR- 2	1 blade of W13	Positive 200 kA, 10/350 μs	24.45	14.19	4.52	398.42	388.28	376.47
SSR- 3	1 blade of W1	Negative 75 kA, 1/200 μs	13.39	8.82	2.79	472.27	461.81	450.31
SSR- 4	1 blade of W13	Negative 75 kA, 1/200 μs	13.39	8.82	2.79	473.11	462.89	451.15
SSR- 5	2 blades of W1	Positive 150 and 150 kA, 10/350 μs	18.57	17.33	4.70	299.36	288.90	277.40
SSR- 6	2 blades of W13	Positive 150 and 150 kA, 10/350 μs	18.57	17.33	4.70	431.56	421.42	409.61
SSR- 7	2 blades of W1	Negative 75 and 75 kA, 1/200 μs	11.15	10.93	2.95	649.95	639.49	627.99
SSR- 8	2 blades of W13	Negative 75 and 75 kA, 1/200 μs	11.15	10.39	2.95	655.62	645.40	633.67
SSR- 9	3 blades of W1	Positive 200,150 and 100 kA, 10/350 μs	24.48	21.84	5.49	406.63	396.18	384.67
SSR- 10	3 blades of W13	Positive 200,150 and 100 kA, 10/350 μs	24.48	21.84	5.49	499.10	499.86	511.63
SSR- 11	3 blades of W1	Negative 100,75 and 50 kA, 1/200 μs	13.98	13.86	3.38	901.11	890.66	879.16
SSR- 12	3 blades of W13	Negative 100,75 and 50 kA, 1/200 μs	13.98	13.86	3.38	914.59	904.37	892.64

### Double sided ring topology

Testing cases for DSR topology were re-made in the same sequence as previous topologies. The parameters of tested cases are given in Table 4 for cases DSR-1 to DSR-12. The results show the DSR topology follows the same features previously introduced in other topologies. Also, feature F3 had the same pattern such that the double and triple blade hits allowed a 22.5% to 95.2% increase in overvoltage when compared to single strikes of the same parameters for phase B of DSR-8 and phase C of DSR-11 respectively. For the last feature introduced in SSR topology where the overvoltages were reduced in it, the DSR also furtherly reduces the overvoltages to be lower than SSR and radial topologies. The percentage of reduction of the maximum overvoltage with respect overvoltages recorded for radial topologies in the same cases were in range of 49.7% for phase A in DSR-11 to 66.7% for phase A in DSR-12.

**Table 4:** Maximum overvoltages recorded for DSR topology.

Case name	Number of blades and the turbine hit	Type and parameters of lightning	Maximum Voltage at (MV)			Maximum Voltage at low voltage bus of grid-connected transformer (kV)		
			blade	Top of tower	Generator	Phase A	Phase B	Phase C
DSR- 1	1 blade of W1	Positive 200 kA, 10/350 μs	24.45	14.19	4.52	241.82	231.30	219.98
DSR- 2	1 blade of W13	Positive 200 kA, 10/350 μs	24.45	14.19	4.52	253.40	256.23	267.83
DSR- 3	1 blade of W1	Negative 75 kA, 1/200 μs	13.39	8.82	2.80	470.43	459.83	448.59
DSR- 4	1 blade of W13	Negative 75 kA, 1/200 μs	13.39	8.82	2.80	189.46	198.33	209.92
DSR- 5	2 blades of W1	Positive 150 and 150 kA, 10/350 μs	18.57	17.33	4.70	298.21	287.62	276.37
DSR- 6	2 blades of W13	Positive 150 and 150 kA, 10/350 μs	18.57	17.33	4.70	288.71	278.45	279.63
DSR- 7	2 blades of W1	Negative 75 and 75 kA, 1/200 μs	11.15	10.93	2.95	647.40	636.80	625.56
DSR- 8	2 blades of W13	Negative 75 and 75 kA, 1/200 μs	11.15	10.93	2.95	232.74	242.99	254.57
DSR- 9	3 blades of W1	Positive 200,150 and 100 kA, 10/350 μs	24.47	21.84	5.49	405.05	394.46	383.22
DSR- 10	3 blades of W13	Positive 200,150 and 100 kA, 10/350 μs	24.47	21.84	5.49	354.24	343.98	332.41
DSR- 11	3 blades of W1	Negative 100,75 and 50 kA, 1/200 μs	13.98	13.85	3.39	897.56	886.96	875.72
DSR- 12	3 blades of W13	Negative 100,75 and 50 kA, 1/200 μs	13.98	13.85	3.39	299.95	310.20	321.78

### Star topology

**Fig 5:**
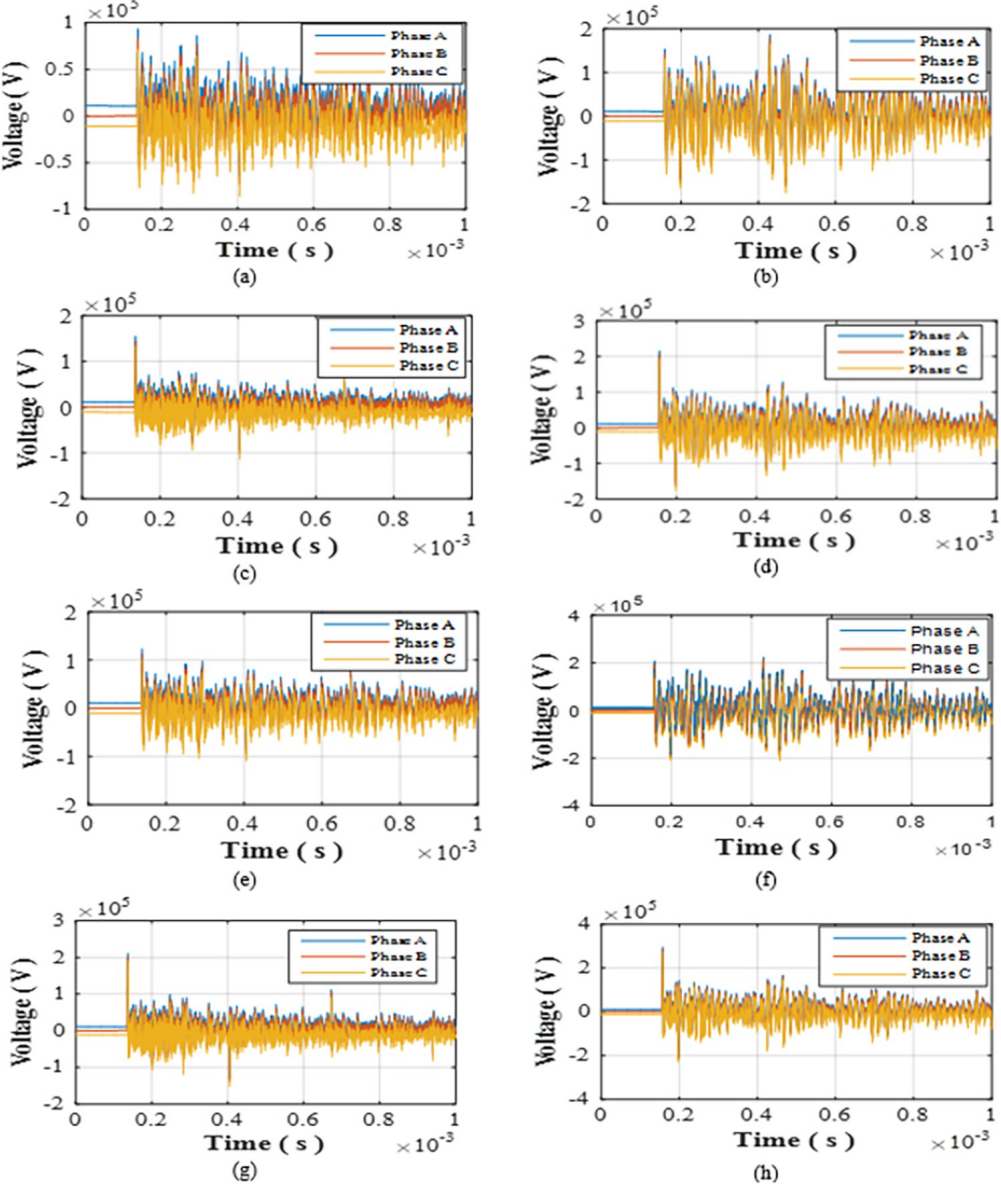
Overvoltages at low-voltage bus of the grid connected transformer in star topology for cases (a) S-5 (b) S-6 (c) S-7 (d) S-8 (e) S-9 (f) S-10 (g) S-11 (h)S-12.

The testing cases for this topology following the same parameters as presented in Table 5. The notation of the cases within this topology will be initiated with letter S from S-1 to S-12. The recorded overvoltage at the low voltage side of the grid connected transformer for cases S5 to S12 are shown in Fig 5. The results of the table confirm the applicability of features F1 to F4 on the star topology. In regards of feature F3, the double and triple blade hits allowed a 16.8% to 103.1% increase in overvoltage when compared to single strikes of the same parameters for phase A of S-5 and phase C of S-11 respectively. The star topology shows the minimal injected overvoltage in comparison to SSR, DSR and radial topologies. The percentage of reduction ranged from 66.7% for phase A in case S-8 to 89.04% for phase C in case S-3. It could be concluded that the star topology has the highest ability to reduce overvoltage with respect to radial topology thus allowing the injected overvoltage reaching the grid to be minimal.

**Table 5:** Maximum overvoltages recorded for star topology.

Case name	Turbine hit	Type and parameters of lightning	Maximum Voltage at (MV)			Maximum Voltage at low voltage bus of grid-connected transformer (kV)		
			blade	Top of tower	Generator	Phase A	Phase B	Phase C
S- 1	1 blade of W1	Positive 200 kA, 10/350 μs	24.45	14.25	4.61	79.87	70.38	69.76
S- 2	1 blade of W13	Positive 200 kA, 10/350 μs	24.45	14.14	4.46	162.84	154.21	151.41
S- 3	1 blade of W1	Negative 75 kA, 1/200 μs	13.39	8.99	3.04	115.08	104.63	93.12
S- 4	1 blade of W13	Negative 75 kA, 1/200 μs	13.39	8.72	2.62	159.66	149.33	141.56
S- 5	2 blades of W1	Positive 150 and 150 kA, 10/350 μs	18.65	17.41	4.79	93.32	82.86	85.87
S- 6	2 blades of W13	Positive 150 and 150 kA, 10/350 μs	18.48	17.25	4.63	186.23	177.60	174.64
S- 7	2 blades of W1	Negative 75 and 75 kA,1/200 μs	11.38	11.17	3.15	154.89	144.44	132.93
S- 8	2 blades of W13	Negative 75 and 75 kA, 1/200 μs	10.95	10.79	2.80	215.23	204.90	193.27
S- 9	3 blades of W1	Positive 200,150 and 100 kA, 10/350 μs	24.48	21.95	5.57	123.44	113.00	108.76
S- 10	3 blades of W13	Positive 200,150 and 100 kA, 10/350 μs	24.48	21.74	5.43	221.68	213.05	210.82
S- 11	3 blades of W1	Negative 100,75 and 50 kA, 1/200 μs	14.32	14.18	3.56	211.10	200.65	189.13
S- 12	3 blades of W13	Negative 100,75 and 50 kA, 1/200 μs	13.73	13.72	3.29	295.13	284.80	273.18

### Analysis and discussion

The variance between the responses of the topologies to lightning strikes could be articulated within the features previously defined in simulation results section. The degree of variance in some features and the peculiar characteristics found in some features are analyzed in this section.

**F1:** it is observed in this feature that the injected overvoltages into the grid from negative lightning strikes are higher from positive strikes even for positive strikes for higher peaks. This is explainable on the bases of the fundamental laws of electromagnetic induction. As the lightning surge is transmitted to generator of the wind turbine through electromagnetic induction between nacelle body and the generator of the turbine. The most effective element of induction is the rate of change of the associated magnetic flux, which is much higher in negative strikes that much lower rise and tail times.
**F2:** positive strikes hitting the farthest turbine overvoltage injects higher overvoltage to the grid than closer ones. This could be explained for radial topology of Fig 1A and generalized for other topologies. As the strikes hits the farthest turbine, the injected overvoltages propagates on a path towards the grid. The path depends on the impedance of the cables which are equal allowing maximum transfer of the travelling wave. The connected transformer for the turbines on that path absorb negligible amount of the surge for their lower impedance during positive strikes which could be considered to have lower frequency than negative strikes. On the other hand, when the positive strike hits turbine closest to the grid, at the first turbine the surge is diverted between two paths, one towards the farm and the other towards grid. The division allows a significant reduction in injected overvoltages to grid in case of hitting the closest turbines than hitting the farthest turbine.
  For negative strikes, the feature is reversed where closest turbine injects higher overvoltages than farthest turbines. The same explanation used in previous paragraph could be re-used with one main fundamental difference, which is the impedance of transformers connected to the turbines. As in negative strikes, the frequency dependent impedance of these transformers will be much higher for negative strikes that have much smaller rise times than for positive strikes. So, when negative strikes hit the farthest turbine and as surge propagates towards the grid, a high potion of the travelling wave will be absorbed by higher impedance transformers. Thus the surge reaches the grid will be much smaller than for positive strikes. Hence, in negative strikes this feature is opposite to positive strikes.
**F3:** This feature could be summarized that hitting multi-blade either double-blade and triple blade had increased the injected overvoltage for all topologies in the following manner. The increase was in range of 20% up to 94%, 23.3% to 97.8%, 22.5% to 95.2% and 16.8% to 103.1% for radial, SSR, DSR and star topologies respectively. Generally, it could be concluded that for all topologies there is an expected increase of 15% to 100% in the injected overvoltage to the grid when the lightning strikes multi-blades.
**F4**: The final feature shows that the star topology had the least injected overvoltage to the grid. This is attributed to its star connection allowing the deviation of injected overvoltage among the highest number of paths and reducing the injected overvoltage through the grid path. The percentage of reduction for the injected overvoltages with respect to radial topology, reached 50.78%, 66.07% and 89.04% for SSR, DSR and star topology respectively. It confirms that the star topology had the highest percentage of reduction in overvoltage compared to radial topology.

The previous comparison could be summarized in a single table as presented in Table 6.

**Table 6:** Comparison of all four topologies.

	Injected Overvoltage to the grid in			
	Radial Topology	SSR Topology	DSR Topology	Star Topology
Magnitude of Overvoltage	Highest overvoltage this type of strike when compared to SSR, DSR and star topology	Overvoltage reduced by range of 11.75% to 50.78%.	Overvoltage reduced by range of 49.7% to 66.7%,	Overvoltage reduced by range of 66.7% to 89.04%,
Impact of double-blade and triple blade hit lightning strikes.	Increased the magnitude of overvoltage in range of 20% up to 94%,	Increased the magnitude of overvoltage in range of 23.3% to 97.8%	Increased the magnitude of overvoltage in range of 22.5% to 95.2%	Increased the magnitude of overvoltage in range of 16.8% to 103.1%
Effect of Positive and negative strikes	Negative strikes cause higher overvoltage. As negative strikes have higher rate of change allowing a higher overvoltage to be induced.			
Effect of hitting the bale of the farthest turbine vs hitting the blade of the closest turbine	• Positive strikes hitting the farthest turbine overvoltage injects higher overvoltage to the grid than closer ones. • Negative strikes hitting the farthest turbine overvoltage injects higher overvoltage to the grid than closer ones. Detailed explanation is provided in F2. Detailed explanation is provided in F2.			

## Conclusion

The topological impact of wind farms upon the injected overvoltage from lightning strikes for possible single and multi-blade strikes were examined in this paper. The data from a real system were used to simulate a testing environment for various possible cases in the simulation process using ATP software. Four main topologies were considered; radial. SSR, DSR and star topologies. Different types of lightning were included either positive or negative strikes with a consideration for a multi-blade hit scenario. The features of the injected overvoltage to the grid were recorded in each topology. These features were used to define the point of similarities and the range of variances between these topologies. The main features could be summarized as follows:

F1. Negative lightning strikes inject higher overvoltage to the grid due to their higher rate of change in comparison to positive lightning strikes. The higher rate of change of negative lightning strikes increases the rate of change of the associated magnetic flux which is reflected in the induced surge which propagates through the wind farm.
F2. Strikes at the farthest turbine from the grid injects higher overvoltage than strikes to the closest turbine for positive strikes. This was found to be due to the flow path of flow of the surges which depends on the impedance value during the strikes. The opposite is applicable for negative strikes. The reason for the difference between positive and negative strikes in this feature is the frequency dependent impedance of the transformers which has much higher value in negative strikes than positive strikes. That difference in impedance would affect the value of surge reaching the gird.
F3. Lightning strikes hits to multi-blades was found to increase the injected overvoltage for all topologies in range of 15% to 100% with respect to the overvoltage generated by lightning strikes hitting single blade.
F4. The star topology had the least injected overvoltage to the grid. The reason behind that is the nature of the star otology which has several parallel path. The propagating surge of overvoltage that gets divided among these paths which results in a significant reduction in their value. Hence the highest reduction of overvoltage value was found in star topology as 89.04%. The percentage of reduction for the injected overvoltages reached 50.78%, 66.07% and 89.04% for SSR, DSR and star topology respectively and all with respect to radial topology.

It is finally recommended to consider star topology for wind farms to minimize any possible injected lightning overvoltage into the grid.
